# Mutanolysin-Digested Peptidoglycan of *Lactobacillus reuteri* Promotes the Inhibition of *Porphyromonas gingivalis* Lipopolysaccharide-Induced Inflammatory Responses through the Regulation of Signaling Cascades via TLR4 Suppression

**DOI:** 10.3390/ijms25010042

**Published:** 2023-12-19

**Authors:** Donghan Kim, Hanhee Choi, Hyeonjun Oh, Jiyeon Lee, Yongjin Hwang, Seok-Seong Kang

**Affiliations:** 1 Department of Food Science and Biotechnology, College of Life Science and Biotechnology, Dongguk University, Goyang 10326, Republic of Korea; 2 Novalacto Co., Ltd., Daejon 34016, Republic of Korea

**Keywords:** *Lactobacillus reuteri*, peptidoglycan, periodontitis, inflammatory responses

## Abstract

Periodontitis is an oral infectious disease caused by various pathogenic bacteria, such as *Porphyromonas gingivalis*. Although probiotics and their cellular components have demonstrated positive effects on periodontitis, the beneficial impact of peptidoglycan (PGN) from probiotic *Lactobacillus* remains unclear. Therefore, our study sought to investigate the inhibitory effect of PGN isolated from *L. reuteri* (LrPGN) on *P. gingivalis*-induced inflammatory responses. Pretreatment with LrPGN significantly inhibited the production of interleukin (IL)-1β, IL-6, and CCL20 in RAW 264.7 cells induced by *P. gingivalis* lipopolysaccharide (LPS). LrPGN reduced the phosphorylation of PI3K/Akt and MAPKs, as well as NF-κB activation, which were induced by *P. gingivalis* LPS. Furthermore, LrPGN dose-dependently reduced the expression of Toll-like receptor 4 (TLR4), indicating that LrPGN inhibits periodontal inflammation by regulating cellular signaling cascades through TLR4 suppression. Notably, LrPGN exhibited stronger inhibition of *P. gingivalis* LPS-induced production of inflammatory mediators compared to insoluble LrPGN and proteinase K-treated LrPGN. Moreover, MDP, a minimal bioactive PGN motif, also dose-dependently inhibited *P. gingivalis* LPS-induced inflammatory mediators, suggesting that MDP-like molecules present in the LrPGN structure may play a crucial role in the inhibition of inflammatory responses. Collectively, these findings suggest that LrPGN can mitigate periodontal inflammation and could be a useful agent for the prevention and treatment of periodontitis.

## 1. Introduction

Probiotics play a crucial role in modulating host immune responses through direct interactions with epithelial and immune cells, offering various health benefits [1,2]. The cell wall components of probiotics are vital molecules that interact with host receptors and regulate immune signaling pathways, resulting in their beneficial effects [3]. Most probiotics are Gram-positive lactic acid bacteria, which are characterized by a cell wall containing a thick peptidoglycan layer associated with proteins, teichoic acid, lipoteichoic acid, and lipoprotein [4]. The peptidoglycan (PGN) within the cell wall is composed of glycan chains that consist of repeating units of *N*-acetylglucosamine (NAG) and *N*-acetylmuramic acid (NAM) and these glycan chains are linked by pentapeptide chains through their N-terminus to NAM [4]. Muramyl dipeptide (MDP) is a minimal bioactive motif of PGN, composed of one carbohydrate and two amino acids [5]. A report has demonstrated that PGN from lactobacilli, as well as MDP, inhibited interleukin (IL)-12 produced by macrophages [6]. Thus, MDP is a pivotal component of PGN in regulating immune responses [7].

Extensive evidence from in vitro and in vivo studies indicates that the cell wall components of probiotics contribute to the modulation of the immune system. For instance, surface layer proteins of *Lactobacillus plantarum* have been shown to confer anti-inflammatory activity in acute colitis mice [8]. Previous studies have also demonstrated that different immunomodulating properties of probiotic strains may be attributed to distinct cell wall molecules or structures. For example, the anti-inflammatory activity of *L. plantarum* K8 against poly I:C-induced IL-8 production was attributed to lipoteichoic acid in the cell wall components, whereas the lipoprotein and PGN of the bacterium did not inhibit IL-8 production [9]. Furthermore, lipoteichoic acid of lactobacilli, such as *L. rhamnosus,* did not suppress IL-8 production [9]. Additionally, *Lactobacillus* PGN has been linked to the enhancement of innate immune responses. Huang et al. [10] reported that PGN of *L. rhamnosus* MLGA increases β-defensin expression and significantly reduces the production of pro-inflammatory cytokines, such as IL-1β, induced by lipopolysaccharide.

Periodontitis is a chronic oral disease that impacts the structures supporting the teeth, including the gingiva [11]. A previous study reported that approximately 42% of adults in the United States suffer from periodontitis, with 7.8% of cases being severe [12]. The significant and increasing global burden of severe periodontitis has grown over the past three decades. In 2019, 1.1 billion people suffered from severe periodontitis globally [13]. Moreover, periodontitis has been shown to have a significant association with severe systemic conditions, including Alzheimer’s disease and rheumatism [14,15]. Chronic periodontitis is caused by multiple pathogenic bacteria, including *Porphyromonas gingivalis* [16]. The lipopolysaccharide of *P. gingivalis* is recognized as a key pathogenic factor of periodontitis and continuously stimulates host immune cells, specifically monocytes and macrophages. In turn, this leads to the destruction of periodontal tissues by releasing inflammatory mediators through Toll-like receptor (TLR)-mediated inflammatory signaling pathways [17,18,19].

Certain probiotics isolated from the oral cavity, including lactobacilli, exhibit antibacterial activities against *P. gingivalis* by producing antimicrobial substances, including bacteriocins, that directly inhibit its growth [20]. Furthermore, many studies have reported that lactobacilli modulate inflammatory responses to periodontal pathogenic bacteria [21,22]. For instance, exposure of gingival epithelial cells to *P. gingivalis* increased the production of IL-1β and tumor necrosis factor (TNF)-α. However, these cytokines were significantly downregulated in the presence of lactobacilli and bifidobacteria [21]. Similarly, *L. heleveticus* LH2171 attenuated the expression of IL-6 and IL-8 in gingival epithelial cells stimulated by *P. gingivalis* [23]. A recent study also demonstrated that bacterial lysates from *L. plantarum* and *L. rhamnosus* GG inhibited IL-1β, IL-6, and MCP-1 production induced by *P. gingivalis* LPS [24]. Postbiotics, specifically probiotic cell wall-associated components, have emerged as promising molecules that exhibit beneficial effects typically attributed to probiotics [25]. However, the mechanisms through which probiotic cell wall-associated components attenuate periodontal inflammation have not been fully elucidated. Therefore, our study sought to investigate the potential inhibitory effects of mutanolysin-digested *L. reuteri* PGN (LrPGN) on *P. gingivalis* LPS-induced inflammatory responses. Furthermore, the inhibitory potential of insoluble *L. reuteri* PGN, which was not treated with mutanolysin (iLrPGN), and proteinase K-treated LrPGN (pLrPGN) was also examined.

## 2. Results

### 2.1. LrPGN Inhibits P. gingivalis LPS-Induced Inflammatory Responses

**Figure 1:**
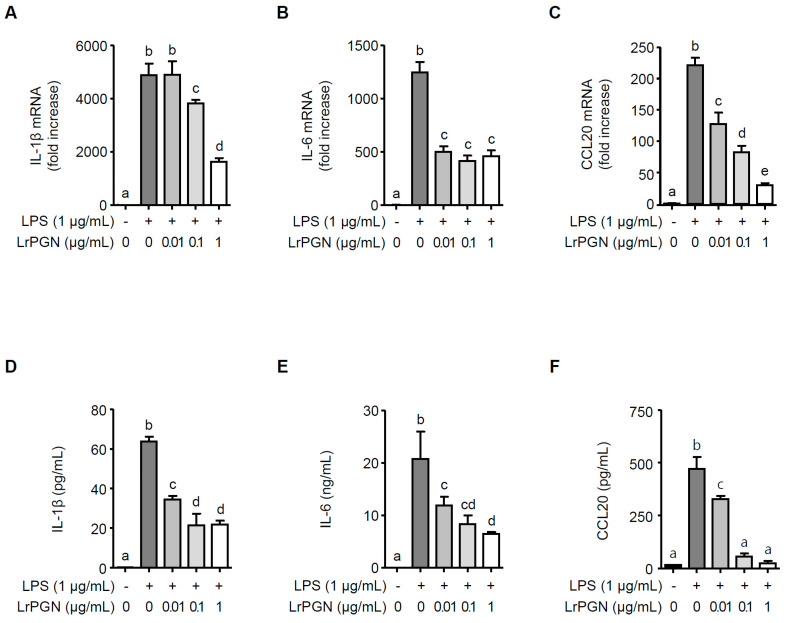
LrPGN inhibits *P. gingivalis* LPS-induced inflammatory responses. RAW 264.7 cells were pretreated with LrPGN (0.01, 0.1, or 1 μg/mL) for 15 h and stimulated with *P. gingivalis* LPS (1 μg/mL) for an additional 3 h. Total RNA was then extracted, and the mRNA expression of IL-1β (**A**), IL-6 (**B**), and CCL20 (**C**) was determined by qRT-PCR. After LrPGN pretreatment as described above, RAW 264.7 cells were stimulated with *P. gingivalis* LPS (1 μg/mL) for an additional 24 h. Spent culture supernatants were collected, and the secretion level of IL-1β (**D**), IL-6 (**E**), and CCL20 (**F**) was assessed using ELISA. The results are expressed as mean ± standard deviation, and statistical significance (*p* < 0.05) was determined by ANOVA. Different letters (a–e) indicate statistical differences between groups.

To examine whether LrPGN inhibits the inflammatory responses induced by *P. gingivalis* LPS in RAW 264.7 cells, the expression of IL-1β, IL-6, and CCL20 was assessed at mRNA and protein levels. As illustrated in Figure 1, *P. gingivalis* LPS significantly increased the expression of IL-1β (Figure 1A), IL-6 (Figure 1B), and CCL20 (Figure 1C) at the mRNA level. However, pretreatment with 0.1 and 1 μg/mL of LrPGN significantly inhibited the mRNA expression of IL-1β (Figure 1A), IL-6 (Figure 1B), and CCL20 (Figure 1C). Furthermore, the lower concentration of LrPGN (0.01 μg/mL) also significantly inhibited *P. gingivalis* LPS-induced IL-6 and CCL20 but not IL-1β. As expected, pretreatment with LrPGN (0.01, 0.1, and 1 μg/mL) dramatically decreased *P. gingivalis* LPS-induced secretion of IL-1β (Figure 1D), IL-6 (Figure 1E), and CCL20 (Figure 1F). These results demonstrate that LrPGN effectively inhibits *P. gingivalis* LPS-induced inflammatory responses.

### 2.2. LrPGN Attenuates the Signaling Pathways of Phosphatidylinositol 3-Kinase (PI3K), Akt, Mitogen-Activated Protein Kinases (MAPKs), and Nuclear Factor-κB (NF-κB) Induced by P. gingivalis LPS

**Figure 2:**
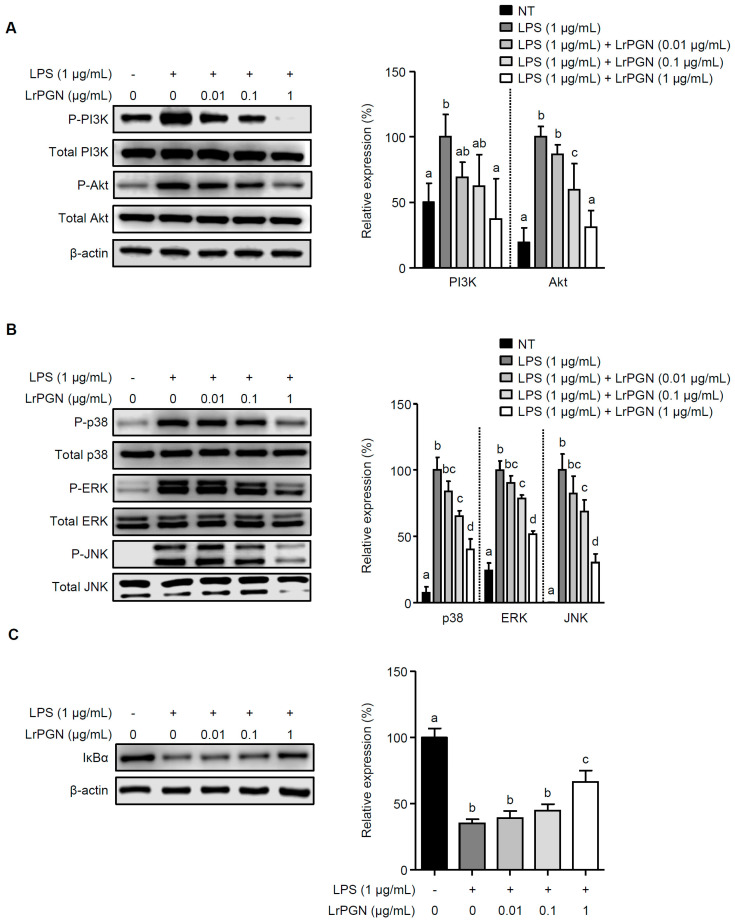
LrPGN attenuates the signaling pathways of PI3K/Akt, MAPKs, and NF-κB induced by *P. gingivalis* LPS. RAW 264.7 cells were pretreated with LrPGN (0.01, 0.1, or 1 μg/mL) for 15 h and stimulated with *P. gingivalis* LPS (1 μg/mL) for 30 min. Phosphorylation of PI3K/Akt (**A**), MAPKs (**B**), and IκBα degradation (**C**) was determined via Western blot analysis. The relative expression of PI3K/Akt, MAPKs, and IκBα is presented as the mean ± standard deviation. Statistical significance (*p* < 0.05) was determined by ANOVA. Different letters (a–d) indicate statistical differences between groups.

We investigated whether LrPGN could attenuate the signaling pathways of PI3K/Akt, MAPKs, and NF-κB in the presence of *P. gingivalis* LPS. *P. gingivalis* LPS alone markedly increased PI3K and Akt phosphorylation, whereas pretreatment with LrPGN decreased PI3K and Akt phosphorylation (Figure 2A). Furthermore, when RAW 264.7 cells were pretreated with LrPGN at the indicated concentrations, followed by *P. gingivalis* LPS stimulation, the phosphorylation of MAPKs, p38, extracellular signal-regulated kinase (ERK), and c-Jun-N-terminal kinase (JNK), was dose-dependently attenuated (Figure 2B). As shown in Figure 2C, *P. gingivalis* LPS alone significantly degraded nuclear factor-κB-inhibitor α (IκBα), suggesting an increase in NF-κB activation. However, pretreatment with LrPGN at concentrations of 0.01 and 0.1 μg/mL gradually restored IκBα degradation, and pretreatment with LrPGN at a higher concentration (1 μg/mL) significantly restored IκBα degradation, indicating NF-κB suppression. These results suggest that LrPGN inhibits *P. gingivalis* LPS-induced inflammatory responses by targeting the signaling pathways of PI3K/Akt, MAPKs, and NF-κB, which are essential for cytokine and chemokine production during inflammation.

### 2.3. LrPGN Exhibits Greater Potential for Inhibiting Inflammatory Responses Induced by P. gingivalis LPS

**Figure 3:**
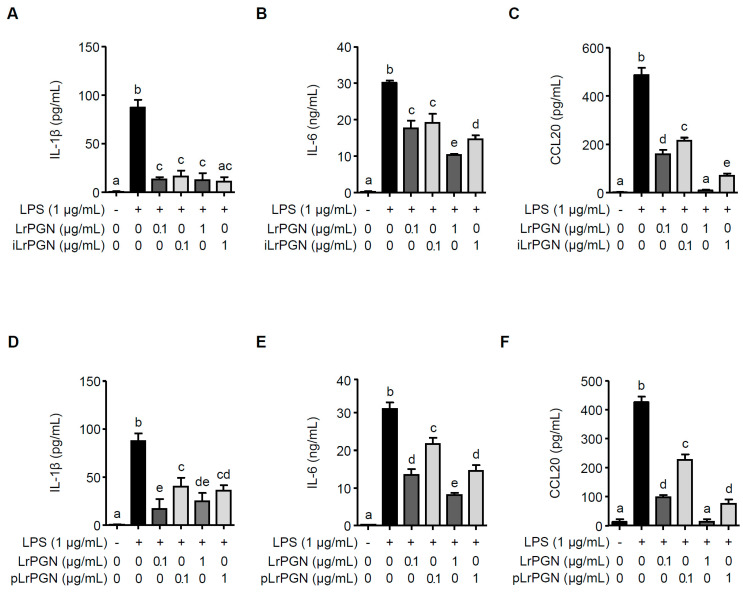
LrPGN exhibits superior potential for inhibiting inflammatory responses induced by *P. gingivalis* LPS. RAW 264.7 cells were pretreated with LrPGN (0.1 or 1 μg/mL) or iLrPGN (0.1 or 1 μg/mL) for 15 h and stimulated with *P. gingivalis* LPS (1 μg/mL) for 24 h. After stimulation, the protein secretion of IL-1β (**A**), IL-6 (**B**), and CCL20 (**C**) was assessed using ELISA. RAW 264.7 cells were pretreated with LrPGN (0.1 or 1 μg/mL) or pLrPGN (0.1 or 1 μg/mL) for 15 h and stimulated as described above. After stimulation, the protein secretion of IL-1β (**D**), IL-6 (**E**), and CCL20 (**F**) was assessed using ELISA. The results are expressed as the mean ± standard deviation, and statistical significance (*p* < 0.05) was determined by ANOVA. Different letters (a–e) indicate statistical differences between groups.

To compare the inhibitory potential of LrPGN, iLrPGN, and pLrPGN, the secretion levels of IL-1β, IL-6, and CCL20 were quantified in *P. gingivalis* LPS-stimulated RAW 264.7 cells with or without pretreatment with LrPGN, iLrPGN, or pLrPGN. As shown in Figure 3A, both LrPGN and iLrPGN at concentrations of 0.1 and 1 μg/mL markedly suppressed the secretion of IL-1β induced by *P. gingivalis* LPS. However, the inhibitory potential of both LrPGN and iLrPGN was not statistically different. Moreover, although iLrPGN at concentrations of 0.1 and 1 μg/mL inhibited *P. gingivalis* LPS-induced IL-6 and CCL20 proteins, the inhibitory potential of LrPGN at 1 μg/mL was greater for IL-6 and CCL20 proteins compared to iLrPGN at 1 μg/mL (Figure 3B and Figure 3C, respectively). When RAW 264.7 cells were pretreated with LrPGN or pLrPGN at 1 μg/mL, both LrPGN and pLrPGN inhibited *P. gingivalis* LPS-induced IL-1β protein secretion, but there was no substantial difference in their inhibitory potential. However, LrPGN pretreatment at 0.1 μg/mL exhibited much stronger inhibition than pLrPGN pretreatment at the same concentration (Figure 3D). Furthermore, LrPGN showed significantly stronger inhibition of *P. gingivalis* LPS-induced IL-6 (Figure 3E) and CCL20 (Figure 3F) protein secretion than pLrPGN at concentrations of 0.1 and 1 μg/mL. These results suggest that the anti-inflammatory potential of LrPGN was greater than that of iLrPGN or pLrPGN.

### 2.4. LrPGN Significantly Suppresses TLR4 mRNA Expression, but Moderately Suppresses TLR2 mRNA Expression

**Figure 4:**
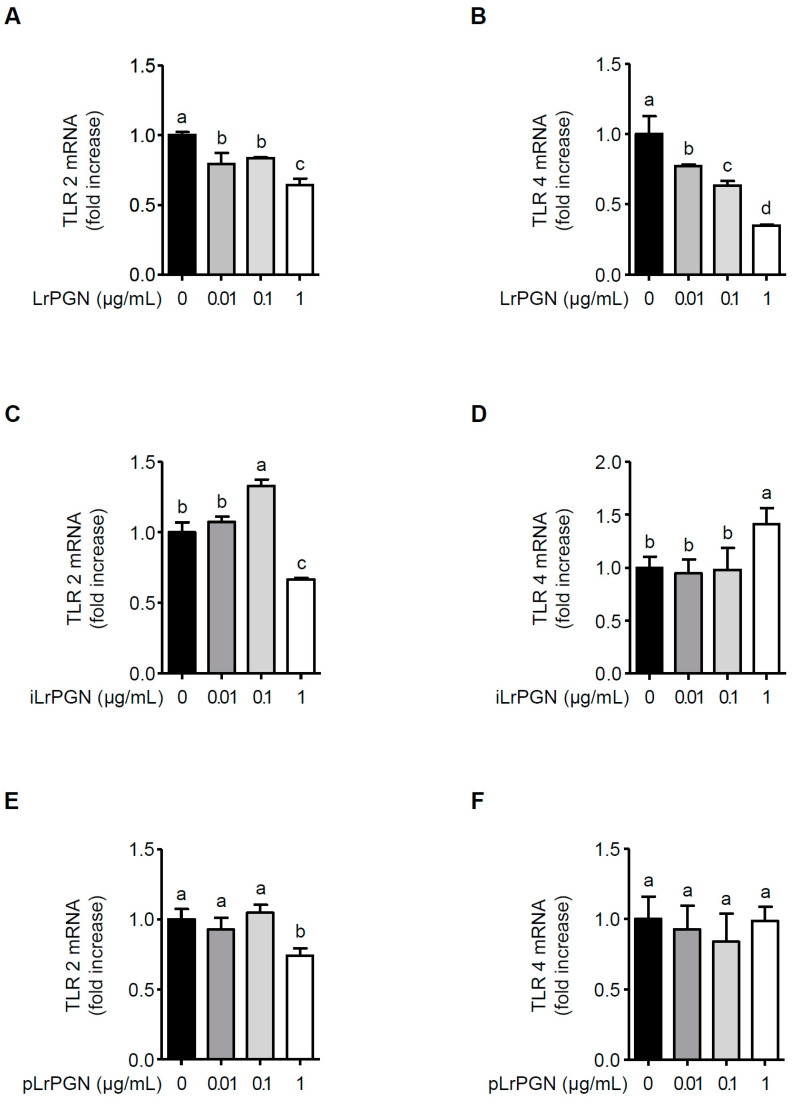
LrPGN significantly suppresses TLR4 mRNA expression. RAW 264.7 cells were pretreated with LrPGN, iLrPGN, or pLrPGN (0.01, 0.1, or 1 μg/mL) for 15 h and stimulated with *P. gingivalis* LPS (1 μg/mL) for 15 h. Total RNA was extracted, and the mRNA expression levels of TLR2 (**A**,**C**,**E**) and TLR4 (**B**,**D**,**F**) were determined by qRT-PCR. The results are expressed as the mean ± standard deviation, and statistical significance (*p* < 0.05) was determined by ANOVA. Different letters (a–d) indicate statistical differences between groups.

We assessed whether LrPGN reduces TLR2 and TLR4 expression. As shown in Figure 4A, LrPGN exhibited a suppressive effect on TLR2 mRNA expression, albeit without a clear dose-dependent manner. Conversely, LrPGN notably and dose-dependently reduced TLR4 mRNA expression (Figure 4B). Notably, while a reduction in TLR2 mRNA expression was observed at a concentration of 1 μg/mL iLrPGN, this effect was not observed in the case of iLrPGN treatment at 0.01 and 0.1 μg/mL for TLR2 mRNA expression (Figure 4C). Additionally, iLrPGN did not inhibit TLR mRNA expression (Figure 4D). Similarly, pLrPGN did not significantly inhibit TLR2 and TLR4 mRNA expression, except when exposed to a concentration of 1 μg/mL pLrPGN (Figure 4E and Figure 4F, respectively). These results imply that LrPGN significantly reduces TLR4 expression and partially reduces TLR2 expression, potentially desensitizing RAW 264.7 cells to *P. gingivalis* LPS and, therefore, inhibiting inflammatory responses.

### 2.5. MDP Significantly Attenuates P. gingivalis LPS-Induced Inflammatory Responses

**Figure 5:**
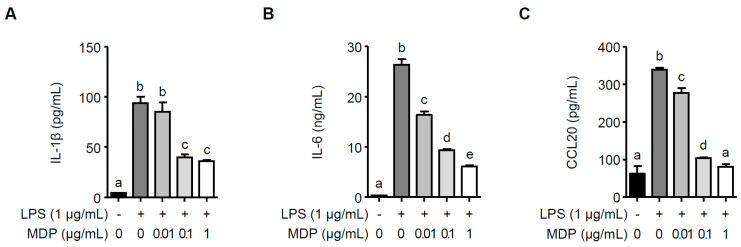
MDP significantly inhibits *P. gingivalis* LPS-induced inflammatory responses. RAW 264.7 cells were pretreated with MDP (0.01, 0.1, or 1 μg/mL) for 15 h and stimulated with *P. gingivalis* LPS (1 μg/mL) for 24 h. After stimulation, the protein secretion of IL-1β (**A**), IL-6 (**B**), and CCL20 (**C**) was assessed using ELISA. The results are expressed as the mean ± standard deviation, and statistical significance (*p* < 0.05) was determined by ANOVA. Different letters (a–e) indicate statistical differences between groups.

Since LrPGN has been shown to attenuate *P. gingivalis* LPS-induced IL-1β, IL-6, and CCL20 in RAW 264.7 cells, we investigated whether MDP is responsible for the inhibition of LrPGN on inflammatory responses induced by *P. gingivalis* LPS. As shown in Figure 5A, pretreatment with 0.1 and 1 μg/mL of MDP significantly reduced *P. gingivalis* LPS-induced IL-1β secretion, whereas the lower concentration of MDP (0.01 μg/mL) did not decrease IL-1β secretion. However, pretreatment with MDP significantly inhibited *P. gingivalis* LPS-induced IL-6 (Figure 5B) and CCL20 (Figure 5C) secretion in a dose-dependent manner. These findings imply that the antagonistic activity of LrPGN against *P. gingivalis* LPS-induced inflammatory responses could be attributed to the presence of MDP in the peptidoglycan structure.

## 3. Discussion

Our findings demonstrated that pretreatment with LrPGN effectively suppressed *P. gingivalis* LPS-induced production of IL-1β, IL-6, and CCL20. The inhibitory effects of LrPGN on inflammatory responses were attributed to the suppression of TLR4 expression, whereas TLR2 expression was only partially reduced. The secretion of various cytokines, including IL-6, has been associated with the PI3K/Akt signaling pathway [26]. Additionally, studies have reported that MAPKs and NF-κB signaling pathways play essential roles in cytokine and chemokine production [24]. IκBs, such as IκBα, play a crucial role in localizing NF-κB in the cytosol, therefore preventing its transcriptional activity [27]. *P. gingivalis* LPS-induced inflammatory responses are mediated by interactions with both TLR2 and TLR4 [28]. The suppression of TLR4 expression further led to the downregulation of the PI3K/Akt, MAPKs, and NF-κB signaling pathways, resulting in the attenuation of *P. gingivalis* LPS-induced inflammatory responses. Moreover, we observed that LrPGN, without mutanolysin treatment and proteinase K-treated LrPGN, showed a slight reduction in inhibitory capacity compared to LrPGN. This suggests that the biological activity of LrPGN could be attributed to MDP-like molecules that activate NOD2. These results emphasize the potential of LrPGN as a promising agent for reducing periodontal inflammation and its potential as an alternative therapeutic option for preventing and treating periodontitis.

Probiotic effector molecules located on the cell surface, such as cell wall components, have been extensively studied for their beneficial effects. Although probiotics offer numerous health benefits, certain limitations, such as strain-specific behaviors, antibiotic resistance, and potential virulence gene transfer, have also been identified [29]. To overcome these constraints, postbiotics have emerged as a novel alternative to probiotics. Postbiotics are cellular components or metabolic products derived from probiotic bacteria that exert beneficial effects on the host [30]. For example, *L. plantarum* lipoteichoic acid (LTA), a major cell wall constituent present in lactic acid bacteria, has been shown to inhibit IL-8 production in Caco-2 cells induced by pam2CSK4, with the inhibitory effect attributed to the lipid and D-alanine moieties of the LTA structure [31]. In our previous study, we demonstrated that LTA isolated from *L. plantarum*, *L. casei*, and *L. rhamnosus* GG suppressed IL-8 production by bacterial flagellin in porcine peripheral blood mononuclear cells [32]. Lipoproteins present in the cell wall of *L. plantarum* WCFS1 exhibited anti-inflammatory properties [33]. Moreover, PGN derived from *L. rhamnosus* and *L. acidophilus* reduced *Escherichia coli* LPS-induced IL-6, IL-1β, and TNF-α secretion and TLR4 expression in RAW 264.7 cells [34]. In contrast, *L. plantarum* PGN did not exhibit inhibitory effects on poly I:C-induced IL-8 production in IPEC-J2 cells [9]. These disparities could be attributed to variations in cell wall components among different *Lactobacillus* species or discrepancies in the stimuli used in the studies. Regardless of these discrepancies, these studies collectively suggest that cell wall components of lactobacilli are typically responsible for suppressing inflammatory responses. More importantly, despite using different *Lactobacillus* strains, our findings were consistent with those of previous studies.

All bacteria, including Gram-positive and Gram-negative bacteria, possess PGN as an essential component for their growth and survival. PGN is composed of repetitive units of β-1,4-linked polymers of NAG and NAM covalently linked to peptide chains comprising 2–5 amino acid residues [35]. Mutanolysin is an enzyme that cleaves the β-1,4 linkage of PGN polymers between NAG and NAM, leading to the release of PGN fragments known as muropeptides [35,36]. Nucleotide-binding and oligomerization domain proteins (NODs) serve as intracellular sensors that recognize PGN fragments [37]. NOD1 recognizes PGN fragments containing D-glutamyl-meso-diaminopimelic acid units, primarily found in Gram-negative bacteria and some Gram-positive bacteria. In contrast, NOD2 recognizes MDP, which is the minimal motif of PGN and is widely distributed in both Gram-positive and Gram-negative bacteria [38]. MDP-related molecules have also been identified in lactic acid bacteria such as *L. salivarius* [39]. PGN fragments are known to enter cells through endocytosis and are recognized by NOD1 or NOD2 receptors, thus triggering intracellular signaling cascades that initiate immunoregulation [35]. Our findings demonstrated that LrPGN, which was digested by mutanolysin, significantly suppressed *P. gingivalis* LPS-induced inflammatory responses. This suppression could potentially be elucidated by the facilitated internalization of LrPGN into cells and the possible interaction of MDP-like molecules of LrPGN within the cytosol. Although LrPGN without mutanolysin, referred to as iLrPGN, also inhibited *P. gingivalis* LPS-induced inflammatory responses, iLrPGN did not exhibit effective anti-inflammatory potential compared to LrPGN. This observation suggests that LrPGN fragments, including MDP-like molecules, released by mutanolysin digestion may be more easily transported into the cell and readily interact with NODs. Additionally, a previous report showed that lysozyme-mediated PGN hydrolysis, which also cleaves β-1,4-glycosidic linkages between NAG and NAM similar to the enzymatic function of mutanolysin, enhances the recognition of PGN by NOD receptors [40]. A previous study also demonstrated that MDP directly binds to NOD2 [5]. Furthermore, our findings demonstrated that MDP effectively suppressed the production of IL-1β, IL-6, and CCL20 induced by *P. gingivalis* LPS. These findings suggest that LrPGN enhances the ability of MDP to enter cells where it is recognized by the NOD2 receptor, therefore inhibiting *P. gingivalis* LPS-induced inflammatory responses. Additionally, MDP-like molecules found in the LrPGN structure could play a crucial role in suppressing inflammatory responses induced by *P. gingivalis* LPS. However, we also observed that proteinase K-treated LrPGN did not exhibit stronger inhibition of *P. gingivalis* LPS-induced inflammatory responses compared to LrPGN. This observation suggests that the degradation of the peptide associated with LrPGN could lead to the loss of its anti-inflammatory activity. Thus, it can be inferred that the presence of MDP or MDP-like molecules in LrPGN is essential to attenuate the inflammatory responses induced by *P. gingivalis* LPS.

We demonstrated that LrPGN effectively suppressed TLR4 expression but only partially affected TLR2 expression. Both TLR2 and TLR4 are pattern-recognition receptors involved in recognizing bacterial cell wall components [41]. Unlike Gram-negative bacteria-derived LPS, which is exclusively recognized by TLR4 to trigger inflammation, *P. gingivalis* LPS can be recognized by both TLR2 and TLR4 [28]. In addition, a previous study demonstrated the effective reduction of TLR4-mediated inflammatory responses by MDP [42]. Similarly, another investigation revealed that silencing NOD2 resulted in an augmentation of TLR4-dependent inflammatory responses [43]. However, there have been conflicting reports regarding the interaction between *P. gingivalis* LPS and TLRs. Zhang et al. [44] demonstrated that TLR4, but not TLR2, is associated with the activation of signaling pathways. Our findings suggest that the inhibition of TLR4 expression by LrPGN may disrupt the inflammatory responses induced by *P. gingivalis* LPS. This disruption could be achieved by inhibiting signaling pathways, including PI3K/Akt, MAPKs, and NF-κB. Previous research has shown that NOD2 signaling can act as an inhibitor of inflammatory responses triggered by TLR4 activation through LPS [45]. Additionally, MDP pretreatment, which activates NOD2, has been demonstrated to reduce the production of TNF-α, IL-1β, and IL-8 in response to TLR4 stimulation [46]. Based on these findings, our study suggests that the reduction of TLR4 expression by LrPGN may be involved in NOD2 activation. However, it is still unclear whether LrPGN directly regulates the expression or activation of NOD2. Further research is thus necessary to elucidate the specific mechanisms through which LrPGN interacts with NOD2 and modulates its activity.

## 4. Materials and Methods

### 4.1. LrPGN Purification

*L. reuteri* KCTC 3594 was purchased from the Korean Collection for Type Cultures (Daejeon, Republic of Korea) and cultured in de Man–Rogosa–Sharpe (MRS) medium (Neogen, Lansing, MI, USA) at 37 °C for 24 h. LrPGN was prepared from *L. reuteri* KCTC 3594 according to previously described protocols [47] with minor modifications. Briefly, after culturing, bacterial pellets were collected via centrifugation and thoroughly washed with 1 M NaCl. The bacterial pellets were subsequently suspended in an extraction buffer (50 mM Trizma base, 0.1 mM EDTA, and 1 mM 2-mercaptoethanol) and mechanically lysed using high-impact zirconium beads (Benchmark Scientific, Sayreville, NJ, USA). After centrifugation, the remaining pellets were resuspended in 0.5% sodium dodecyl sulfate (SDS) and then incubated at 60 °C for 30 min. The pellets were then centrifuged and once again washed thoroughly with phosphate-buffered saline to remove SDS, then resuspended in 5 mL of 1 M Tris-HCl (pH 7.0) supplemented with DNase (Roche Diagnostics, Mannheim, Germany) and RNase (Sigma-Aldrich, St. Louis, MO, USA). The suspension was incubated at 37 °C for 2 h, followed by the addition of 50 mg MgCl_2_ and 1 mg trypsin, after which the suspension was further incubated at 37 °C for 24 h to digest cell wall-bound residual proteins. Next, the residual pellets were collected by centrifugation at 19,000× *g* for 10 min and were treated with 48% hydrochloric acid at 4 °C for 24 h to eliminate teichoic acid and lipoteichoic acid. The resulting pellets were then thoroughly washed with distilled water, lyophilized, and weighed. Afterward, the lyophilized pellets were digested in endotoxin-free water (1 mL) containing 500 U of mutanolysin (Sigma-Aldrich) at 37 °C for 36 h to obtain LrPGN. After inactivating the enzyme via incubation at 100 °C for 10 min, the obtained LrPGN (100 μg/mL) was further treated with 100 μg of proteinase K (Sigma-Aldrich) at 37 °C for 36 h.

### 4.2. Cell Culture

RAW 264.7 cells were obtained from the American Type Culture Collection (Manassas, VA, USA) and cultured in Dulbecco’s modified Eagle’s medium (Welgene, Gyeongsan, Republic of Korea) containing fetal bovine serum (Gibco, Burlington, ON, Canada) and penicillin/streptomycin (HyClone, Logan, UT, USA) in a humidified incubator with 5% CO_2_ at 37 °C.

### 4.3. Reverse Transcription and Quantitative Real-Time Polymerase Chain Reaction (qRT-PCR)

RAW 264.7 cells (1 × 10^5^ cells/mL) were incubated in a 12-well culture plate at 37 °C for 24 h and pretreated with LrPGN (0.01, 0.1, and 1 μg/mL) at 37 °C for 15 h. After pretreating with LrPGN, the cells were stimulated with 1 μg/mL of *P. gingivalis* LPS for an additional 3 h. Total RNA was then extracted using TRIzol reagent (Invitrogen, Carlsbad, CA, USA) according to the manufacturer’s instructions. Complementary DNA (cDNA) was synthesized by mixing the total RNA with random hexamers (Promega, Madison, WI, USA) and M-MLV reverse transcriptase (Promega). Target genes were amplified by RT-PCR in a total volume of 20 μL containing cDNA (2 μL), SYBR green real-time PCR master mix (10 μL) (Toyobo, Osaka, Japan), 10 pmol of forward and reverse gene-specific primers (1 μL), and sterile water (7 μL) using a StepOnePlus^TM^ Real-Time PCR System (Applied Biosystems, Foster City, CA, USA). The PCR amplification protocol consisted of an initial denaturation at 95 °C for 10 s, followed by 40 cycles at 95 °C for 5 s and at 60 °C for 30 s. The specific primers for target genes used in this study are as follows: IL-1β, forward 5′-CTCACAAGCAGAGCACAAGC-3′ and reverse 5′-TCTTGGCCGAGGACTAAGGA-3′; IL-6, forward 5′-TCCTACCCCAATTTCCAATGCT-3′ and reverse 5′-TCTGACCACAGTGAGGAATGTC-3′; CCL20, forward 5′-ATGGCCGATGAAGCTTGTGA-3′ and reverse 5′-CTCCTTGGGCTGTGTCCAAT-3′; TLR2, forward 5′-CTGCGAAGTGGAAACCATCC-3′ and reverse 5′-CTCCTTGGGCTGTGTCCAAT-3′; TLR4, forward 5′-ACTCAGCAAAGTCCCTGATGAC-3′ and reverse 5′-ACTCAGCAAAGTCCCTGATGAC-3′ and reverse 5′-AGTTTGAGAGGTGGTGTAAGCC-3′; β-actin, forward 5′-TACAGCTTCACCACCACAGC-3′ and reverse 5′-GGAAAAGAGCCTCAGGGCAT-3′. The relative mRNA expressions of IL-1β, IL-6, CCL20, TLR2, and TLR4 were normalized to β-actin and calculated using the 2^−ΔΔCt^ method.

### 4.4. Enzyme-Linked Immunosorbent Assay (ELISA)

The protein secretion of IL-1β, IL-6, and CCL20 in RAW 264.7 cells was quantified via ELISA. RAW 264.7 cells (1 × 10^5^ cells/mL) were incubated in a 48-well culture plate at 37 °C for 24 h and pretreated with LrPGN (0.01, 0.1, and 1 μg/mL) for 15 h. The cells were then stimulated with *P. gingivalis* LPS (1 μg/mL) for an additional 24 h, after which the spent culture supernatants were collected. To compare the inhibitory potential of LrPGN, insoluble LrPGN (iLrPGN) that had not been treated with mutanolysin, and proteinase K-treated LrPGN (pLrPGN), RAW 264.7 cells (1 × 10^5^ cells/mL) were pretreated with LrPGN, iLrPGN, or pLrPGN at concentrations of 0.1 and 1 μg/mL for 15 h. In a separate experiment, RAW 264.7 cells (1 × 10^5^ cells/mL) were pretreated with muramyl dipeptide (MDP; Invivogen, San Diego, CA, USA) (0.01, 0.1, and 1 μg/mL) for 15 h. The cells were then stimulated with *P. gingivalis* LPS (1 μg/mL) for an additional 24 h, after which spent culture supernatants were collected. The secretion of IL-1β, IL-6, and CCL20 in the spent culture supernatants was determined using commercial ELISA kits (R&D Systems, Minneapolis, MN, USA) according to the manufacturer’s instructions.

### 4.5. Western Blot Analysis

RAW 264.7 cells (1 × 10^5^ cell/mL) were incubated in a 6-well culture plate at 37 °C for 24 h and pretreated with LrPGN (0.01, 0.1, and 1 μg/mL) for 15 h. Next, the cells were stimulated with *P. gingivalis* LPS (1 μg/mL) for 30 min, and cell lysates were obtained using a lysis buffer (1 M HEPES, 1 M NaCl, 1% IGEPAL^®^ CA-630, 0.75% sodium deoxycholate, and 10% glycerol) containing protease and phosphatase inhibitors. Protein concentrations were determined using a bicinchoninic assay kit (Thermo Fisher Scientific, Waltham, MA, USA). Equal amounts of protein samples were loaded and separated by SDS-polyacrylamide gel electrophoresis and transferred to polyvinylidene difluoride (PVDF) membranes (Millipore, Bedford, MA, USA). After blocking with 5% skimmed milk in Tris-buffered saline containing 0.1% Tween 20 (TBST), the PVDF membranes were incubated with primary antibodies specific to PI3K, phosphorylated PI3K, Akt, phosphorylated Akt, p38, phosphorylated p38, extracellular signal-regulated kinase (ERK), phosphorylated ERK, c-Jun-N-terminal kinase (JNK), phosphorylated JNK, and nuclear factor-κB-inhibitor α (IκBα) (Cell Signaling Technology, Danvers, MA, USA) and β-actin (Santa Cruz Biotechnology Inc., Santa Cruz, CA, USA) at 4 °C overnight. The PVDF membranes were then washed with TBST and probed with horseradish peroxidase-conjugated anti-rabbit IgG (Cell Signaling Technology) or anti-mouse IgG (Santa Cruz Biotechnology) at room temperature for 2 h. The immunoreactive bands were detected using an enhanced chemiluminescence reagent (Dyne Bio, Seongnam, Korea) and quantified using a C-DiGit Blot Scanner (Li-Cor Bioscience, Lincoln, NE, USA).

### 4.6. Statistical Analysis

All experiments were performed independently at least three times. The results were expressed as mean ± standard deviation. Statistical significance was determined by one-way analysis of variance (ANOVA) using the IBM SPSS Statistics 25.0 software (IBM, Armonk, NY, USA). *p* < 0.05 was considered statistically significant.

## 5. Conclusions

In conclusion, our study demonstrated that LrPGN possesses potent anti-inflammatory activity against *P. gingivalis* LPS. Although previous studies have demonstrated the positive effects of cell wall components derived from various lactobacilli, such as LTA, on oral infectious diseases, the beneficial effects of *Lactobacillus* PGN have remained largely unexplored. Our findings suggest that MDP, a partial structure of PGN, plays a crucial role in inhibiting *P. gingivalis* LPS-induced inflammatory responses. Additionally, LrPGN reduces inflammation by suppressing TLR4 through NOD2, thus influencing important signaling cascades. Therefore, LrPGN holds promise as a valuable therapeutic agent for the treatment of periodontitis.

## Data Availability

Data are available from the corresponding author upon request.
